# High-throughput enrichment and isolation of megakaryocyte progenitor cells from the mouse bone marrow

**DOI:** 10.1038/s41598-021-87681-2

**Published:** 2021-04-15

**Authors:** Lucas M. Bush, Connor P. Healy, James E. Marvin, Tara L. Deans

**Affiliations:** 1 Department of Biomedical Engineering, University of Utah, Salt Lake City, UT 84112 USA; 2 Flow Cytometry Core Facility, University of Utah Health Sciences Center, Salt Lake City, UT 84112 USA

**Keywords:** Haematopoiesis, Thrombopoiesis

## Abstract

Megakaryocytes are a rare population of cells that develop in the bone marrow and function to produce platelets that circulate throughout the body and form clots to stop or prevent bleeding. A major challenge in studying megakaryocyte development, and the diseases that arise from their dysfunction, is the identification, classification, and enrichment of megakaryocyte progenitor cells that are produced during hematopoiesis. Here, we present a high throughput strategy for identifying and isolating megakaryocytes and their progenitor cells from a heterogeneous population of bone marrow samples. Specifically, we couple thrombopoietin (TPO) induction, image flow cytometry, and principal component analysis (PCA) to identify and enrich for megakaryocyte progenitor cells that are capable of self-renewal and directly differentiating into mature megakaryocytes. This enrichment strategy distinguishes megakaryocyte progenitors from other lineage-committed cells in a high throughput manner. Furthermore, by using image flow cytometry with PCA, we have identified a combination of markers and characteristics that can be used to isolate megakaryocyte progenitor cells using standard flow cytometry methods. Altogether, these techniques enable the high throughput enrichment and isolation of cells in the megakaryocyte lineage and have the potential to enable rapid disease identification and diagnoses ahead of severe disease progression.

## Introduction

Megakaryocytes (MKs) are important cells within the hematopoietic lineage because they produce platelets, the anucleate cells that facilitate healing. Platelets are released into the bloodstream from MKs where they play a central role in homeostasis, immune responses, and hemostasis^1,2^. Platelets also play an important role in thrombosis, which underlies heart attacks and stroke, making them the target of many pharmaceutics and therapies. MKs differentiate from hematopoietic stem cells (HSCs)^3^, but the mechanisms of MK and subsequent platelet development are not fully understood, leading to difficulties when identifying the circumstances that cause disease^4–7^. MK maturation is characterized by an increase in cell size and DNA content to develop multilobed polyploid nuclei, an upregulation of MK-specific surface markers, and cytoplasmic remodeling to prepare for platelet production^5,8^. A significant challenge with studying MK development, and the diseases that arise from disrupted MK development, is the identification and classification of the progenitor cells that are produced from HSCs and subsequently give rise to MKs^9^. Although MKs are continually produced through adulthood, HSCs and MKs account for only about 0.01% and 0.05–0.1% of cells, respectively, in the mouse bone marrow. The frequency of MK progenitors is expected to be similarly infrequent which presents significant challenges for large-scale studies of this intermediate cell population^10,11^.

Recent studies suggest that MKs are derived from HSCs through multiple parallel lineage pathways^12–15^. While these lineage pathways are not fully elucidated, their diversity highlights the need to identify and isolate MK progenitor cells in order to understand MK development and maturation. However, because the bone marrow contains a heterogenous population of cells, it is difficult to identify MK progenitor cells. Single-cell transcriptomics have provided biological snapshots that depict hematopoietic development as a continuum of differentiation, where a subpopulation of HSCs develops along a trajectory with an intrinsic bias to become platelets^16,17^. These quantitative approaches provide a high-resolution transcriptomic landscape of single timepoints throughout hematopoietic development that have helped to inform more standardized approaches for distinguishing individual cells in this lineage by using a combination of antibodies to recognize cell surface specific markers to identify cells based upon their immunofluorescent profiles when analyzed by flow cytometry. Using this method, cellular subpopulations can be sorted and re-cultured for further investigation.

**Figure 1:**
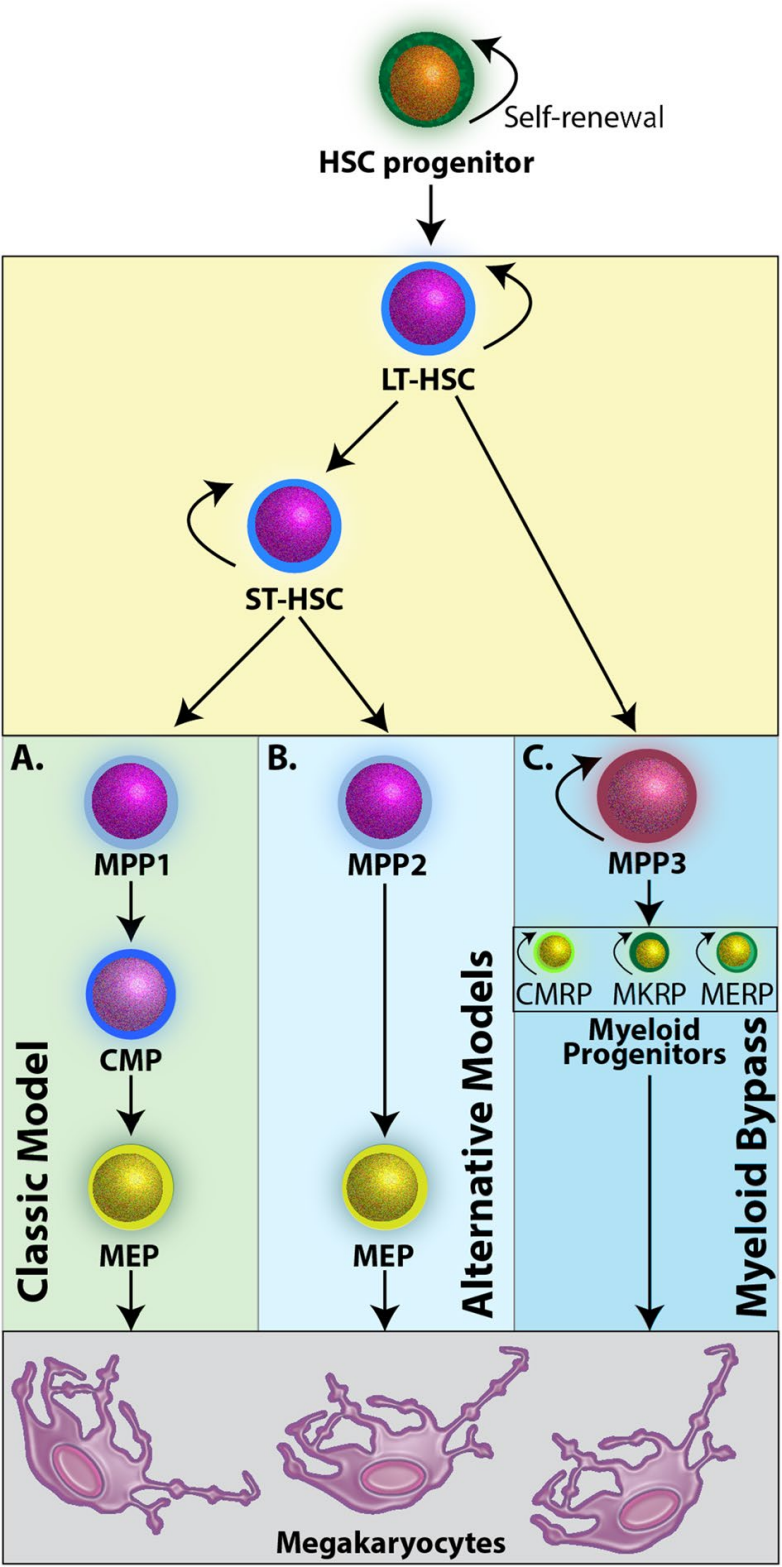
Models of MK differentiation from HSCs. (**A**) In the classic model, cell-fate decisions progress in a step-wise fashion where ST-HSCs differentiate toward an MPP1 cell population that has no self-renewal capacity but can further commit to CMP cells, MEP cells, and finally MKs. (**B**) The alternative model yields MEP cells directly from a non-self-renewing MPP2 cell type. (**C**) The myeloid bypass model suggests that LT-HSCs are primed to directly give rise to an MPP3 cell type capable of producing myeloid progenitor populations that all have the capacity to self-renew and maintain specific cell populations. *HSC* hematopoietic stem cell; *LT-HSC* long-term hematopoietic stem cell; *ST-HSC* short-term hematopoietic stem cell; *MPP* multipotent progenitor; *CMP* common myeloid progenitor; *MEP* megakaryocyte/erythroid progenitor; *MK* megakaryocyte; *CMRP* common myeloid repopulating progenitor; *MKRP* megakaryocyte repopulating progenitor; *MERP* megakaryocyte-erythroid repopulating progenitor.

In the mouse, HSCs are broadly defined by the Lin^-^ Sca-1^+^ c-Kit^+^ (LSK) immunophenotype^18,19^, and have two distinct populations: (1) HSCs that are capable of long-term bone marrow reconstitution (LT-HSCs) and (2) HSCs capable of short-term hematopoietic reconstitution (ST-HSCs). Two different differentiation pathways, classical and alternative, have been proposed where ST-HSCs give rise to MK cells in which precursor cells progress through multiple differentiation states in a step-wise fashion before becoming a MK cell^19^ (Fig. 1A,B). Notably, in neither of these pathways are the MK progenitor cells (MPP1, MPP2, respectively) thought to have any proliferative potential. In contrast, the recently proposed myeloid bypass model suggests that a population of self-renewing multipotent progenitor cells (MPP3), which are formed directly from LT-HSCs, give rise to an MK repopulating progenitor (MKRP) population of cells^15,20^ (Fig. 1C). The myeloid bypass model suggests that the formation and development of MKs may be dynamic and sensitive to the ever-changing biological environments present during inflammation, infection, and wound healing^15,21^. Identifying and isolating this MKRP cell population holds the potential to rapidly advance our understanding of MK development and maturation.

Because most cells develop distinct morphological characteristics at various stages of differentiation, we hypothesize that cellular morphology may be an important parameter to identify the MKRP cell population. Consistent with this hypothesis, commitment down the MK lineage is characterized by an increase in cell size, endoreplication that increases DNA content, and cytoplasmic remodeling to prepare for platelet production^5,8^. These individual morphological changes, along with fluorescently-labeled cell markers, can be observed using image flow cytometry, where an image of each individual cell is captured as it flows through the cytometer^22^. Each captured image contains data of multiple variables that can be used to characterize individual cells by cell size, shape, and membrane texture, as well as expression levels of fluorescently-labeled cell surface markers. These characteristics can be quantified using computational approaches like principal component analysis (PCA). PCA is a statistical method that groups highly correlated data, such as phenotypic characteristics, by converting a set of possibly correlated variables in a multivariate dataset into sets of values called principal components. Principal components allow multidimensional observations to be grouped based upon overall similarity in order to identify trends in datasets that would otherwise be intractable due to their high dimensionality. In short, this method captures variance in a multivariant dataset to identify the most correlated features that can be used to distinguish cells from different stages of lineage development. Here, we couple conventional antibody labeling, image flow cytometry, and PCA analysis to identify a repopulating population of MK progenitor cells. By coupling these analyses with a cell culture method that enriches for MKRP cells we can reliably identify these cells from heterogenous bone marrow samples using both image flow and conventional flow cytometry. We envision that this will facilitate studies leading to a better understanding the development of MKRP cells, in addition to approaches for increasing MK and platelet production for disease treatment.

## Results

### Image flow cytometry analysis of bone marrow cells following TPO-mediated expansion

MKRP cells often express canonical MK markers that are similar to other myeloid cells, making it difficult to distinguish early progenitor subpopulations from other hematopoietic cells without using large antibody panels. Since most cells develop distinct morphological differences when they are at various stages of cell fate, cell morphology is an additional parameter that can be used to identify MKRP cells. Image flow cytometry is an ideal tool for identifying cell populations and distinct stages of lineage development based upon these differences because it enables the simultaneous analysis of morphologic characteristics and cell surface marker expression.

**Figure 2:**
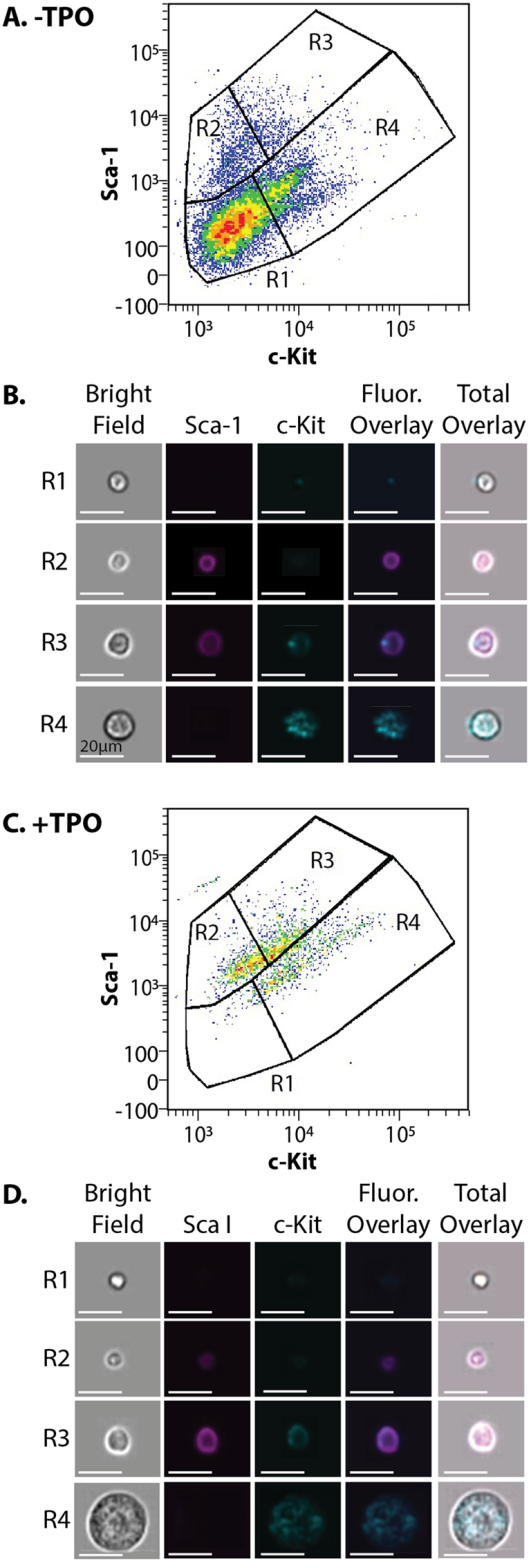
Characterization of progenitor cell subpopulations of mouse bone marrow cells. (**A**) Cells were harvested from the mouse bone marrow (− TPO), stained with LSK antibodies, and run on an image flow cytometer. Cells were gated for Lin^-^ cells and the total population was divided into four distinct subpopulations (R1–R4). (**B**) As cells ran through the image flow cytometer, an image was captured of each cell. Representative images of the cells (− TPO) in each of each of the four subpopulations are represented. (**C**) Bone marrow cells grown in the presence of TPO (+ TPO), stained with LSK antibodies and run on the image flow cytometer. Cells were divided into four distinct subpopulations (R1–R4). (**D**) Representative images of the cells (+ TPO) panels of each of the four subpopulations are represented. Scale bars for all images, 20 μm.

To identify the cells that participate in the commitment to MK cells, mouse bone marrow cells were harvested and characterized by the expression of c-Kit, Sca-1, and lineage (Lin) surface markers. This immunofluorescence labeling strategy, known as LSK staining (Lin^-^ Sca-1^+^ c-Kit^+^), is historically used to identify HSCs that can be distinguished by analysis using flow cytometry. The TPO/c-Mpl signaling within the hematopoietic niche of the mouse bone marrow is essential for HSC survival, proliferation and differentiation into mature MKs^5,8,12,23–26^. Therefore, the TPO cytokine is commonly used for stimulating and differentiating HSCs to become MKs^27^. In all experimental groups, the − TPO condition are cells harvested directly from the bone marrow, then immediately prepared for analysis. In the + TPO condition, bone marrow cells are harvested and grown in TPO for 72 h prior to analysis. Cells in the − TPO condition were initially stained with LSK antibodies and run on the image flow cytometer (Fig. 2A). After culturing with TPO, the + TPO cells were also stained with LSK antibodies and run on the image flow cytometer. The + TPO cells experience a shift in Lin, Sca-1, and c-Kit expression (Fig. 2C), indicating these cells are primed for differentiation. In both the − TPO and + TPO conditions, four subpopulations of cells were defined (R1–R4) based on their Sca-1 and c-Kit expression. The boundaries for these subpopulations were defined by confirming cell surface labeling in the captured images. For example, all cells in the R2 gate were confirmed to be positive for only Sca-1, R3 for both Sca-1 and c-Kit, R4 for only c-Kit, and all cells in the R1 gate were negative for both Sca-1 and c-Kit labeling (Supplementary Figs. S1-S3). Comparing image files associated with the cells in each condition shows drastic morphological changes of the cells in the R4 subpopulation of cells (Fig. 2B,D). Cells in the R4 subpopulation are substantially larger and have increased in Sca-1 and c-Kit expression after exposure to TPO. These cells are present in both growth conditions and enrichment upon TPO treatment suggests that TPO may prime the cells in the R4 subpopulation for lineage commitment into MKs.

### Principal component analysis identifies MKRP cells

**Figure 3:**
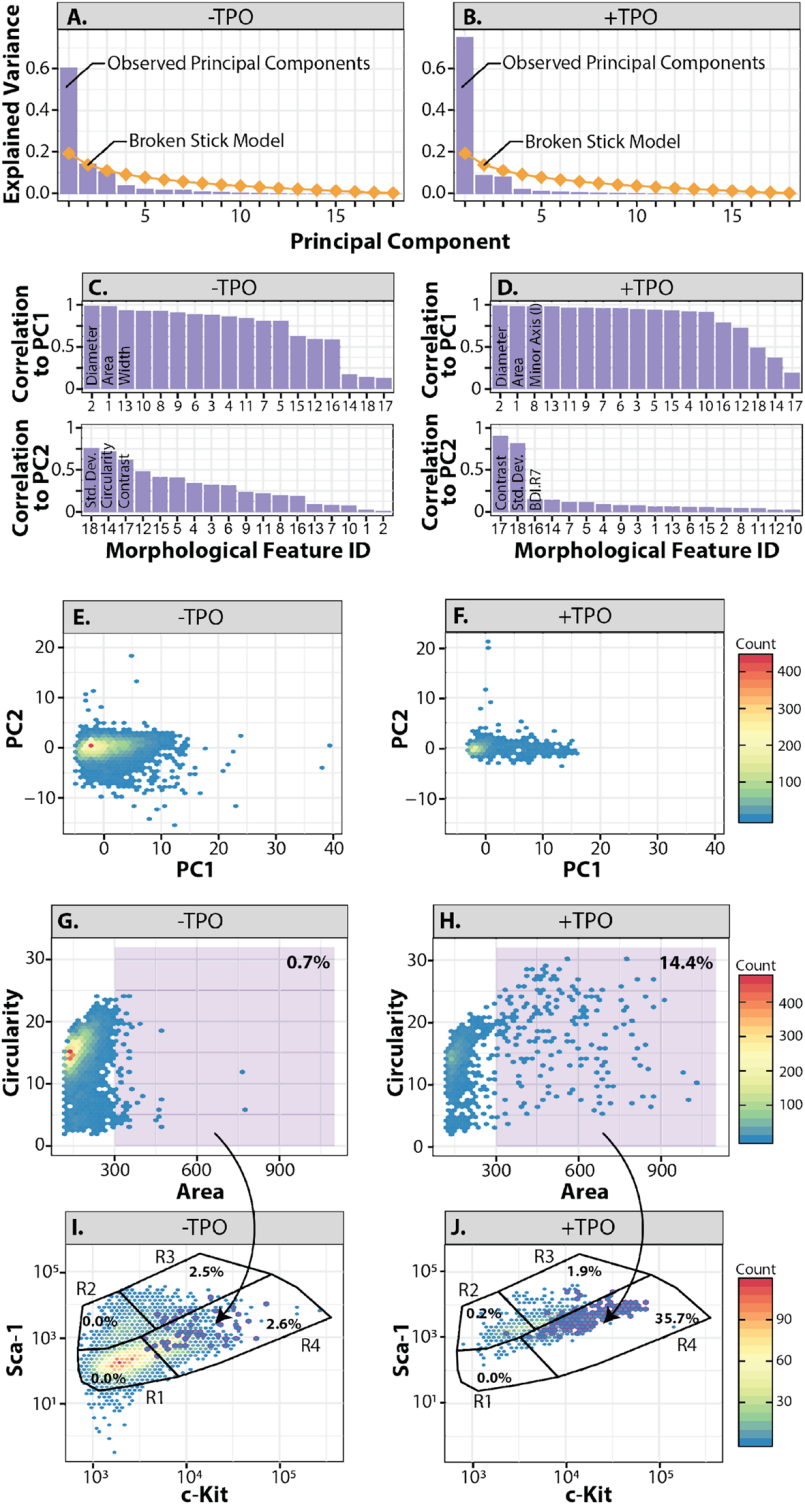
Morphology predicts MKRP phenotype using PCA. (**A,B**) Scree plots showing percent eigenvalue for each principal component in the LSK morphology dataset in the (**A**) − TPO and the (**B**) + TPO conditions. The theoretical percent variation under the broken-stick model is represented as a diamond dashed line, which indicates the boundary between interpretable and uninterpretable principal components. (**C,D**) The absolute contribution/correlation of each morphological feature to each morphological principal component in the (**C**) − TPO and (**D**) + TPO conditions. (**E,F**) Projections of LSK cells grown in the (**E**) −TPO and (**F**) + TPO conditions on the space defined by the first and second principal components. (**G,H**) Cell area vs. circularity of LSK cells in the (**G**) − TPO and the (**H**) + TPO conditions. A gate is included (shaded purple) to mark particularly large and circular cells with the percent of cells present in each gate. (**I,J**) PCA data mapped back onto the experimental flow data in the (**I**) − TPO and (**J**) + TPO conditions.

Because HSCs have been shown to differentiate into MK cells in the presence of TPO, we aimed to use PCA to identify transitions in cell morphologies to locate the MKRP cells. To validate that distinct subpopulations of cells can be characterized morphologically, PCA was used to mathematically capture the variance in image datasets obtained from the image flow cytometer for both the − TPO and + TPO conditions stained with the LSK immunofluorescence panel. With PCA, the input variables contribute to each principal component such that the first principal component explains the largest possible variance in the dataset^28^. The closer a given cell’s principal component score is to another cell’s principal component score, the more similar those two cells are morphologically. This approach determined the most correlated morphological characteristics of each cell using the bright field images for − TPO cells (Fig. 2B) and + TPO (Fig. 2D), respectively. Twenty-nine morphological features corresponding to the shape, size, and texture of the cells in the dataset were considered in the principal component analysis (Supplementary Fig. S4A-E). To determine which principal component to keep, each principal component was compared to the broken stick model, which is a probabilistic model that determines the principal components needed to effectively interpret the data. When using the broken stick model, any principal component that exceeds the model indicates that principal component should be used for further analysis. This comparison showed that the − TPO dataset, PC1 (size) explained 60% of the morphological variation between cells while PC2 (shape) explained 14% of the morphological variation between cells, both exceeding the broken stick model (Fig. 3A)^29^. In the + TPO dataset, only PC1 (size) exceeded the broken stick model but PC1 also explained over 75% of the morphological variation in the dataset (Fig. 3B). Therefore, TPO treatment resulted in a population of cells that were more similar in shape, but more variable in size. In both datasets, PC1 correlated most highly with the size parameters: diameter and area (Fig. 3C,D). This suggests that the morphology of cells in both datasets varied most widely based on these two categories.

Projecting the principal component scores of each cell from the − TPO and + TPO conditions onto a plot shows that TPO treatment reduces morphological variation due to shape (PC2) while enhancing morphological variation due to size (PC1) (Fig. 3E,F). Furthermore, the remaining variation in the + TPO dataset is primarily due to differences in size (Fig. 3F). Overall, PCA revealed that cell size, particularly in + TPO populations, may be sufficient to distinguish cells in the R4 subpopulation. To test this, plots of cell area versus circularity were generated and gating was applied to cells with an area greater than 300 (Fig. 3G,H). By plotting the cells identified in this gate back onto the flow plots, we demonstrate that TPO treatment increases the size of a specific population of cells and that this population belongs primarily to the R4 subpopulation of cells identified in the LSK stain image flow cytometry dataset (Fig. 3I, J). Because MK maturation is marked by an increase in cell size^5^, and high levels of c-Kit expression indicates an intrinsic MK bias^30^, we hypothesized that the R4 subpopulation of cells is a LT-HSC-derived MKRP subset of cells capable of self-renewal and differentiating into mature MKs.

### Flow cytometry analysis of MKRP cells

**Figure 4:**
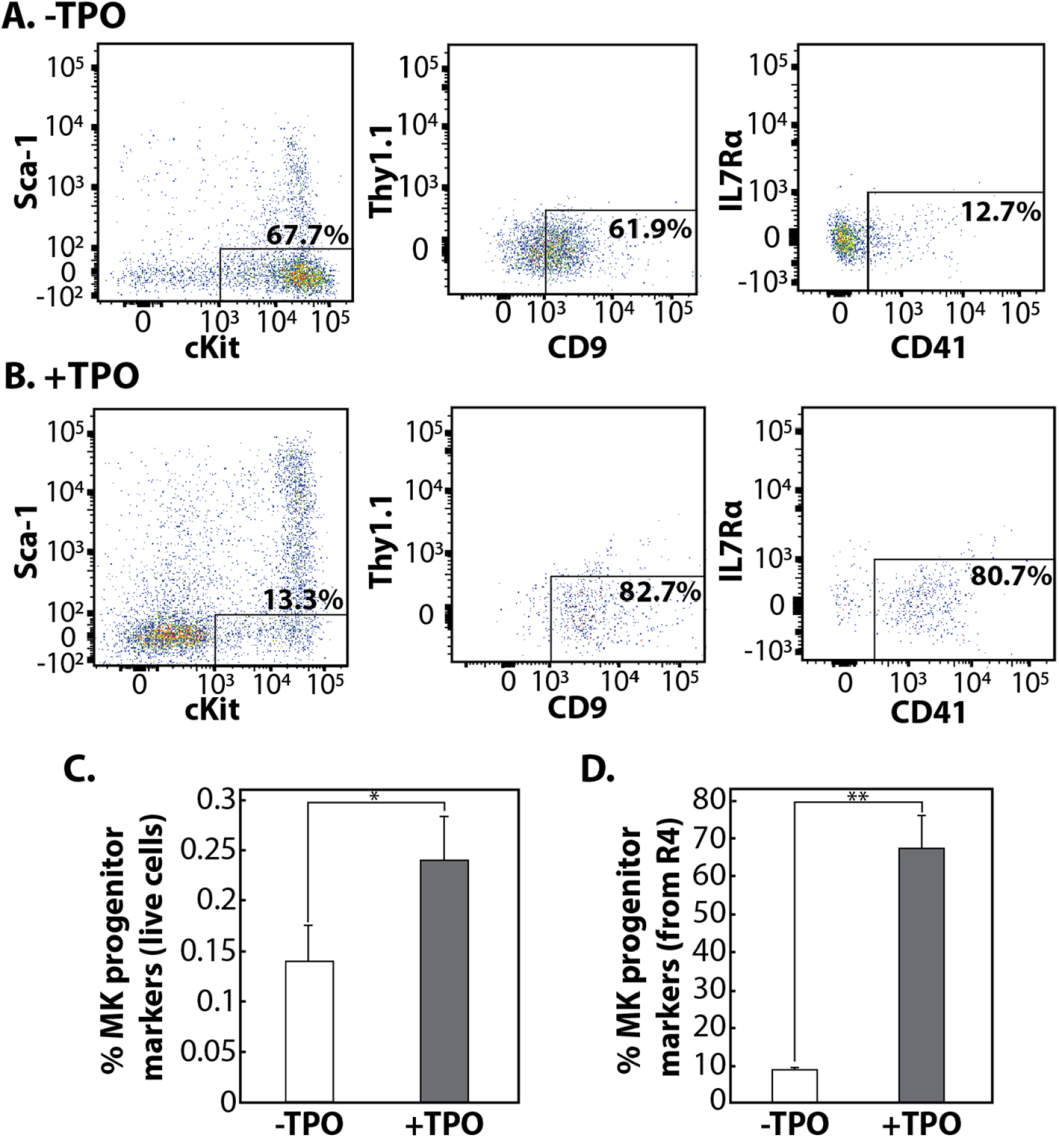
A subset of bone marrow cells are MKRP cells. (**A**) Representative flow cytometry panel of − TPO cells after gating on live, lineage negative cells. Boxes represent gates to identify the potential MKRP cells. (**B**) Representative flow cytometry panel of + TPO cells after gating on live, lineage negative cells. Boxes represent gates to identify the potential MKRP cells. (**C**) The percentage of live cells that stain for MKRP cell markers in the −TPO and + TPO groups, respectively. (**D**) The percentage of R4 cells (Lin^−^Sca1^−^cKit^+^) that stain for MK progenitor cell markers in the − TPO and + TPO groups, respectively. For (**C**) and (**D**) the percent of specified cells represents the average from three independent mice. The error bars represent the standard deviation of the mean. *p < 0.05; **p < 0.005.

To investigate the PCA prediction that MKRP cells exist in the R4 subpopulation, cells from the − TPO and + TPO conditions were stained with antibodies to identify MKRP cells^12,31,32^ using flow cytometry (Fig. 4A,B). Initially, a gate was drawn based on the Lin, Sca-1, and c-Kit expression to identify the R4 subpopulation of cells identified by the PCA analyses as being the potential MKRP population of cells in both growth conditions (Fig. 4A,B). To determine if this population of cells is undergoing MK differentiation and are of HSC origin, a gate was drawn to include CD9 positive and Thy1.1 negative cells. To confirm that these cells were committing to the MK lineage and not the B-cell lineage, a gate was drawn to include cells that were IL7Rα negative and CD41 positive (Fig. 4A,B). Comparing the percentage of MKRP cells in each condition, we showed that a subset of the R4 subpopulation contains MKRP cells that can be enriched when cultured with TPO (Fig. 4C,D).

### Image flow cytometry analysis of MK lineage subpopulations.

**Figure 5:**
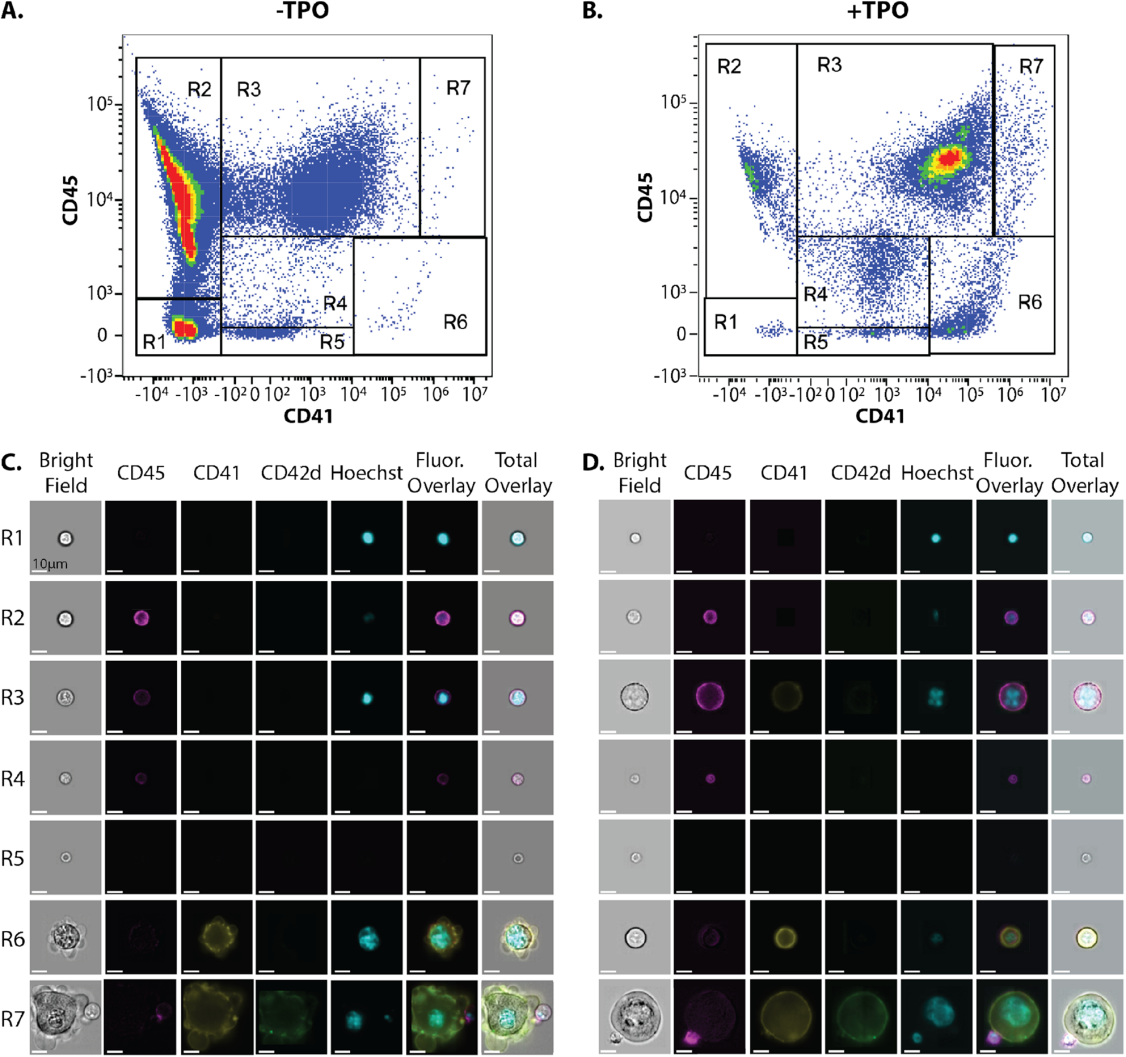
Classification of MK lineage subpopulations. (**A,B**) Flow cytometry plots displaying CD45 and CD41 intensity of bone marrow cells stained with MK antibodies in the (**A**) − TPO and (**B**) + TPO conditions. Seven distinct subpopulations were identified (R1–R7). (**C**) Representative images of cells in the − TPO and (**D**) + TPO conditions. Scale bars, 10 μm for all images.

To further classify the potential of the MKRP cell population, we investigated the development and maturation of these cells. To observe the transition from HSCs to MKs, mouse bone marrow cells from the − TPO and + TPO conditions were stained with MK antibodies (Supplementary Table S1) and image flow cytometry was performed to assess the expression of the MK surface markers (Fig. 5A,B)^33^. In both experimental cases, flow cytometry analysis revealed seven subpopulations of cells (R1–R7) with distinct CD45 and CD41 expression levels (Fig. 5A,B, Supplementary Fig. S5). In the + TPO condition, the cell subpopulations shifted (Supplementary Fig. S6) and increased in MK surface marker expression levels, indicating cells differentiating from a multipotent state HSC state to a more committed MK state.

Qualitative inspection of image files associated with individual flow cytometric events led to the observation that cells in the R7 subpopulation of cells were exceptionally large with the co-expression of MK surface markers and multilobed, polyploid nuclei within the confines of the cell membrane (Fig. 5C,D). To quantify this, the mean fluorescence intensity (MFI) of the three MK surface markers and the total area of the Hoechst 33342 signal were calculated for each subpopulation, revealing that the subpopulation of cells in the R7 gate expressed the highest levels of all three surface markers and had the largest nuclear area of the seven subpopulations (Supplementary Fig. S7). When plotting brightfield circularity versus bright field area, the R7 population was easily distinguishable from the other subpopulations in both culture conditions (Supplementary Fig. S8), further confirming the R7 subpopulation as mature MKs.

### Enrichment and isolation of a repopulating MKRP progenitor cell population

**Figure 6:**
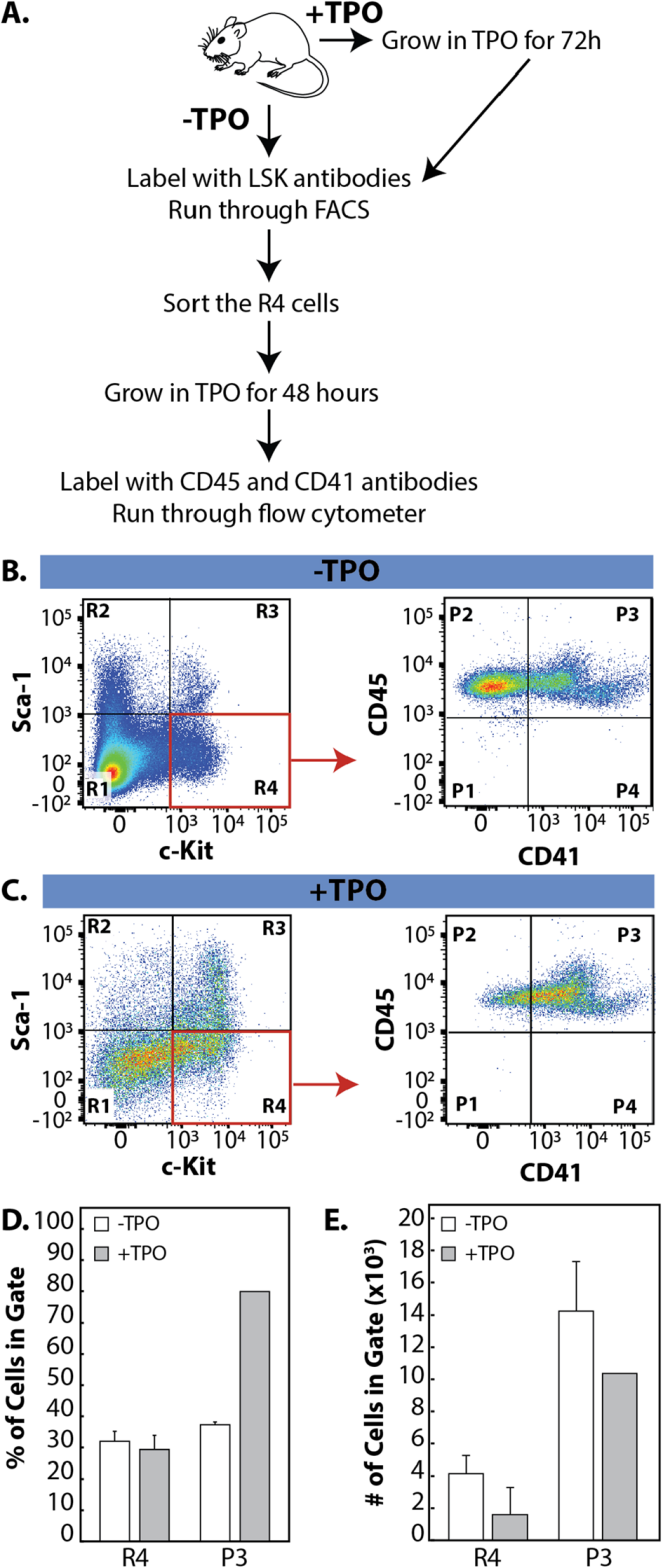
Identification of a repopulating MKRP cell population. (**A**) Overview of experimental design. Bone marrow cells were harvested and split into two populations: 1. − TPO cells were labeled with LSK antibodies, run on the FACS and the R4 subpopulation was sorted, then grown in TPO for 48 h before labeling with the MK cell markers, CD45 and CD41. 2. + TPO cells were grown in TPO for 72 h, labeled with LSK antibodies, run on the FACS, and the R4 subpopulation was sorted then regrown in TPO for an additional 48 h before staining with the MK cell markers CD45 and CD41. (**B**) Flow cytometry plot of − TPO cells stained with LSK antibodies. The defined subpopulations were identified (R1–R4). R4 cells were sorted (red box) and regrown in TPO for an additional 48 h, then stained for MK markers and run on the flow cytometer. New subpopulations were identified (P1–P4) by the expression of MK surface markers. (**C**) Flow cytometry plot of + TPO cells stained with LSK antibodies to identify the defined subpopulations (R1–R4). R4 cells were sorted (red box) and regrown in TPO for an additional 48 h then stained for MK markers and run on the flow cytometer. New subpopulations were identified (P1–P4) by the expression of MK surface markers. (**D**) Percentage of cells in the R4 (MKRP cells) and P3 (MKs) gates. (**E**) Number of cells in the R4 (MKRP cells) and P4 (MKs) gate. For (**D**) and (**E**) error bars represent the standard deviation of the mean from three independent bone marrow harvests from mice.

The ability to identify and isolate the MKRP cell population in an easy and high throughput fashion would significantly improve studies on MK and platelet development. Since our PCA analysis strongly suggested that the R4 subpopulation of LSK cells potentially contain a repopulating MK progenitor cell population, we directly tested this using standard fluorescent activated cell sorting (FACS). The defined R4 subpopulation of cells from the − TPO condition were sorted using FACS, then grown with TPO for 48 h, and labeled with MK antibodies prior to analysis on the flow cytometer (Fig. 6A, − TPO). The defined R4 subpopulation of cells from the + TPO group were also sorted using FACS, and grown with TPO for an additional 48 h. The + TPO cells were labeled with MK antibodies and the expression of these cell surface markers were assessed on the flow cytometer (Fig. 6A, + TPO). In the − TPO group, it was found that 30% of cells were c-Kit^+^ (Fig. 6 B, D), consistent with our previous results. When the R4 cells from the − TPO group were sorted using FACS, then cultured with TPO for an additional 48 h, the cells continued to proliferate, however, only 30% of the cells differentiated into MKs (Fig. 6B,D). Also consistent with previous results, 30% of cells in the + TPO group remained c-Kit^+^ after the first 72 h of culture with TPO (Fig. 6C,D). However, 80% of the R4 cells from the + TPO group differentiated into MKs (Fig. 6C,D) while continuing to proliferate (Fig. 6E). These data demonstrate that cells pre-treated with TPO had a much higher propensity to differentiate into mature MKs compared to the cells not pre-treated with TPO, and indicates TPO pre-treatment as a method for the identification and enrichment of an MKRP cell population capable of self-renewal.

## Discussion

The classic model of hematopoiesis has been challenged over the last several years, transitioning away from the traditional, sequential differentiation pathway toward one that includes branches, bypassing and feedback loops^12,13,18,25,34^. One of the most highly debated aspects of the continuously evolving hematopoietic cell lineage organization is the origin and development of unipotent MKRP cells capable of giving rise to mature MKs. LT-HSCs and MKs have many features in common; most appreciably are their mutual dependence on TPO/c-Mpl stimulation and surface marker expression^5,8,18,23–26,35^. As a result, drawing the line between LT-HSCs, MKRP cells, and mature MKs to systematically study these cells has been exceedingly difficult. Previous methods have lacked the ability to image cells while simultaneously assessing the surface marker expression in response to culture conditions, ultimately limiting analytical capabilities. This study coupled cell surface antibody labeling and image flow cytometry to collect morphology data that is intrinsically coupled with surface marker expression to identify elusive MKRP cell types that can be further isolated to study the mechanisms of MK maturation and platelet development.

By combining mathematical approaches with experimental design, we identified, enriched, and isolated a population of MKRP cells. Subsequent characterization of these cells revealed that the c-Kit^+^Sca-1^−^Lin^−^ fraction of cultured bone marrow cells was highly enriched for unipotent LT-HSC-derived MKRP cells as defined by the CD9^+^ CD41^+^ c-Kit^+^ Sca-1^−^ IL7Ra^-^ Thy1.1^-^Lin^-^ immunotype. Furthermore, sorted MKRP cells that had been previously exposed to TPO retained the ability to self-renew and readily differentiate into mature MKs when re-cultured in TPO, similar to platelet-biased HSCs or stem-like MK progenitor cells identified in other studies^20,21^. Because these cells continue to proliferate when recultured with TPO after FACS, they can be considered a LT-HSC that has gone through the myeloid bypass pathway of differentiation to become MKRP cells.

Altogether, this study validates the use of minimal antibody panels and simple cell culture techniques for the enrichment and isolation of MKRP cells. Applying these methods to the disease setting may improve insights to the early stages of MK commitment and development. Additionally, these methods can be used to better understand the molecular mechanisms underlying atypical MK and platelet development to improve targeted therapies for diseases that arise from skewed cell fate decisions. Finally, this work sets the stage to better understand the role of dynamic gene expression patterns during the development of MKs and platelets to inform alternative approaches for directing stem cell fate that may include reprogramming progenitor cells with gene circuits to drastically increase the production of MKs and platelets *in vitro*^36–39^.

## Materials and methods

The methods and experimental protocols in this manuscript were carried out in accordance with ethics approval from the University of Utah under Institutional Animal Care and Use Committees (IACUC) approved guidelines. The use of animals and/or animal-derived materials in this study were carried out in accordance with relevant ARRIVE and University of Utah guidelines and regulations.

### Bone marrow dissection and cell culture

Mice were euthanatized by CO_2_ asphyxiation, followed by cervical dislocation based upon IACUC approved guidelines. Femurs were dissected from the mouse, excess tissue was removed, and the epiphyses were snipped off. Bone marrow was flushed with IMDM (ThermoFisher Scientific, #12440053) + 10% FBS (Gibco, #16000044) and 1X Penicillin–Streptomycin (ThermoFisher Scientific, #15140122) using a 26.5-gauge needle and collected in a 35 mm dish. Red blood cells were lysed using ACK Lysis Buffer (ThermoFisher Scientific, # A1049201) for 5 min at room temperature, then the solution was centrifuged at 300 × g for 5 min. The cells were washed 2 × with HBSS (Gibco, #14025092) then counted using a hemocytometer. If cells were cultured before analysis, 1 × 10^7^ cells were incubated in IMDM + 10% FBS supplemented with 50 ng/mL of recombinant murine thrombopoietin (TPO) (PeproTech, #315–14) in a T75 flask at 37 °C in 5% CO_2_ for 72 h.

### Preparation for image flow cytometry

After direct isolation from the bone marrow (− TPO) or culture with TPO for 72 h (+ TPO), cells were washed 2 × with PBS. For the LSK stain, cells were resuspended in PBS + 3% BSA (ThermoFisher Scientific, #BP1600-100) with the addition of PE-Cy7 Rat anti-Mouse CD117, PE Rat Anti-Mouse Ly-6A/E, and APC Mouse Lineage Antibody Cocktail, and incubated on ice protected from light for 30 min. Cells were washed 2 × with PBS and resuspended at a final concentration of 2 × 10^7^ cells/ml in PBS. DAPI was added at least 5 min prior to running the sample on the image flow cytometer.

For the MK stain, cells were resuspended in prewarmed IMDM + 10% FBS containing Hoechst 33342 solution, then incubated at 37 °C for 45 min. Cells were washed with PBS then resuspended in PBS + 3% BSA with the addition of PE-Cyanine7 CD45 Monoclonal Antibody, PE Rat Anti-Mouse CD41, and APC CD42d Monoclonal Antibody and incubated on ice, protected from light for 30 min. Cells were washed 2 × with PBS then resuspended at a final concentration of 2 × 10^7^ cells/mL in PBS. 7-AAD was added at least 5 min prior to running the sample on the image flow cytometer. For a complete description of staining reagents, see Supplementary Table S1.

### Image flow cytometry data acquisition and analysis

All experiments used the ImageStreamX Mark II Imaging Flow Cytometer and all image flow cytometry analysis was performed in the IDEAS Software. Data from image flow cytometry experiments was acquired by gating for events that were in focus, did not contain calibration focus beads, and excluded viability dyes. For a full description of data acquisition and analysis methods, consult the INSPIRE ImageStream X Mark II Imaging Flow Cytometer user’s manual and the IDEAS software user manual available online. Gating strategies used in all experiments are described in the Supplemental Information (Supplementary Figs. S1,S5).

### Image analysis and morphological feature extraction

With the LSK stain dataset, brightfield images of lineage-negative cells from each experimental group (− TPO and + TPO, respectively) were first isolated from the full dataset. Individual cells in each image were distinguished from the background and defined automatically using the default brightfield settings in the IDEAS software to produce masked images. Next, the IDEAS software’s built-in feature analysis utility was applied to the masked images and used to measure various morphological features of each cell. In total, twenty-nine morphological features were computed for each cell. Thirteen size features were computed: area; diameter, height; length; major/minor axis; major/minor axis weighted by intensity; perimeter; spot area min; thickness min/max; and width. Next, ten shape features were computed: aspect ratio; intensity weighted aspect ratio; circularity; compactness; elongatedness; lobe count; shape ratio; two-lobe symmetry (symmetry 2); three lobe symmetry (symmetry 3); and four lobe symmetry (symmetry 4). Lastly, six texture features were computed: the intensity of bright details with radii less than 3 (BDI.R3) and radii less than 7 (BDI.R7); contrast; modulation; spot count; and standard deviation. Full descriptions of these features and the means by which they are computed can be found in the IDEAS software user manual. Finally, each observed cell was assigned a categorical variable, R1, R2, R3, or R4, based on the previously specified gates for Sca-1 and c-Kit expression. For the surface marker expression analysis, each cell was assigned its corresponding Sca-1 and c-Kit expression level instead of a categorical variable.

### Principal component analysis

The variance of all twenty-nine morphological features was computed and those with variances less than 0.1 were omitted from further analysis (Supplementary Fig. S4). These features were aspect ratio intensity, aspect ratio, compactness, elongatedness, lobe count, shape ratio, symmetry 2–4, modulation, and spot count. The remaining eighteen morphological features from 31,265 observations in the − TPO dataset were centered, and scaled/normalized such that the values of each feature ranged from 0 to 1 (i.e. min–max normalization). Next, the principal components of the normalized dataset were computed using R’s **prcomp** function^40^. The number of principal components needed to describe each dataset was determined using the broken stick model and David Zelený ‘s **evplot** function^41,42^. Observed principal components that describe more variance than the amount of variance predicted under the broken-stick model are considered necessary to effectively interpret the data. In this experiment, it was determined that the first two principal components were needed to adequately describe the data. Finally, bi-plots (plots with principal components on each axes) and correlation plots were generated with the **ggplot2** library^35^. These steps were repeated for the + TPO dataset to generate the biplots (Fig. 3 E, F). For the surface marker expression bi-plots, the color of each observation was determined based on the relative expression of the surface markers c-Kit (green) and Sca-1 (blue) (Fig. 6, Supplementary Fig. S4). The dynamic range of this color map was defined to capture 99% of the observations. These same steps were repeated to generate the principal components for the 10,926 observations from the + TPO dataset.

### Fluorescence activated cell sorting (FACS), re-culture, and antibody staining of the MKRP subpopulation

For the − TPO group, cells were directly isolated from the mouse bone marrow and prepared for FACS using LSK antibodies as described above. For the + TPO group, cells were isolated from the mouse bone marrow and cultured with 50 ng/mL of TPO for 72 h, then prepared for FACS using LSK antibodies as described above. Cells were then sorted into IMDM + 10% FBS with the addition of 50 ng/mL TPO using the BD FACSAria III, plated into a cell culture dish, and incubated at 37 °C for an additional 48 h. After 48 h, cells were stained with PE-Cyanine7 CD45 monoclonal antibody and PE rat anti-mouse CD41 and run on the BD FACSCanto II. All BD FACSAria III and BD FACSCanto II data analysis was performed using FlowJo.

## Supplementary Information

Supplementary Information.

## Data Availability

Data in used in the PCA in this manuscript were obtained from the ImageStream X Mark II Imaging flow cytometer, as detailed in the Methods section. We will freely share data and code upon request.
