# Supplementary material for: High-throughput enrichment and isolation of megakaryocyte progenitor cells from the mouse bone marrow
Source: Sci Rep. 2021 Apr 15;11:8268. doi: 10.1038/s41598-021-87681-2 (PMC8050096; doi:10.1038/s41598-021-87681-2)
Supplement: Supplementary file 1 — Supplementary Information. [file 41598_2021_87681_MOESM1_ESM.docx]

**SUPPLEMENTARY INFORMATION**

**High-throughput enrichment and isolation of megakaryocyte progenitor cells from the mouse bone marrow**

Lucas M. Bush^1^, Connor P. Healy^1^, James E. Marvin^2^ and Tara L. Deans^1*^

^1^Department of Biomedical Engineering, University of Utah, Salt Lake City, UT 84112, USA

^2^Flow Cytometry Core Facility, University of Utah Health Sciences Center, Salt Lake City, UT 84112, USA

^*^Corresponding author: [tara.deans@utah.edu](mailto:tara.deans@utah.edu)

**Supplementary Figures**


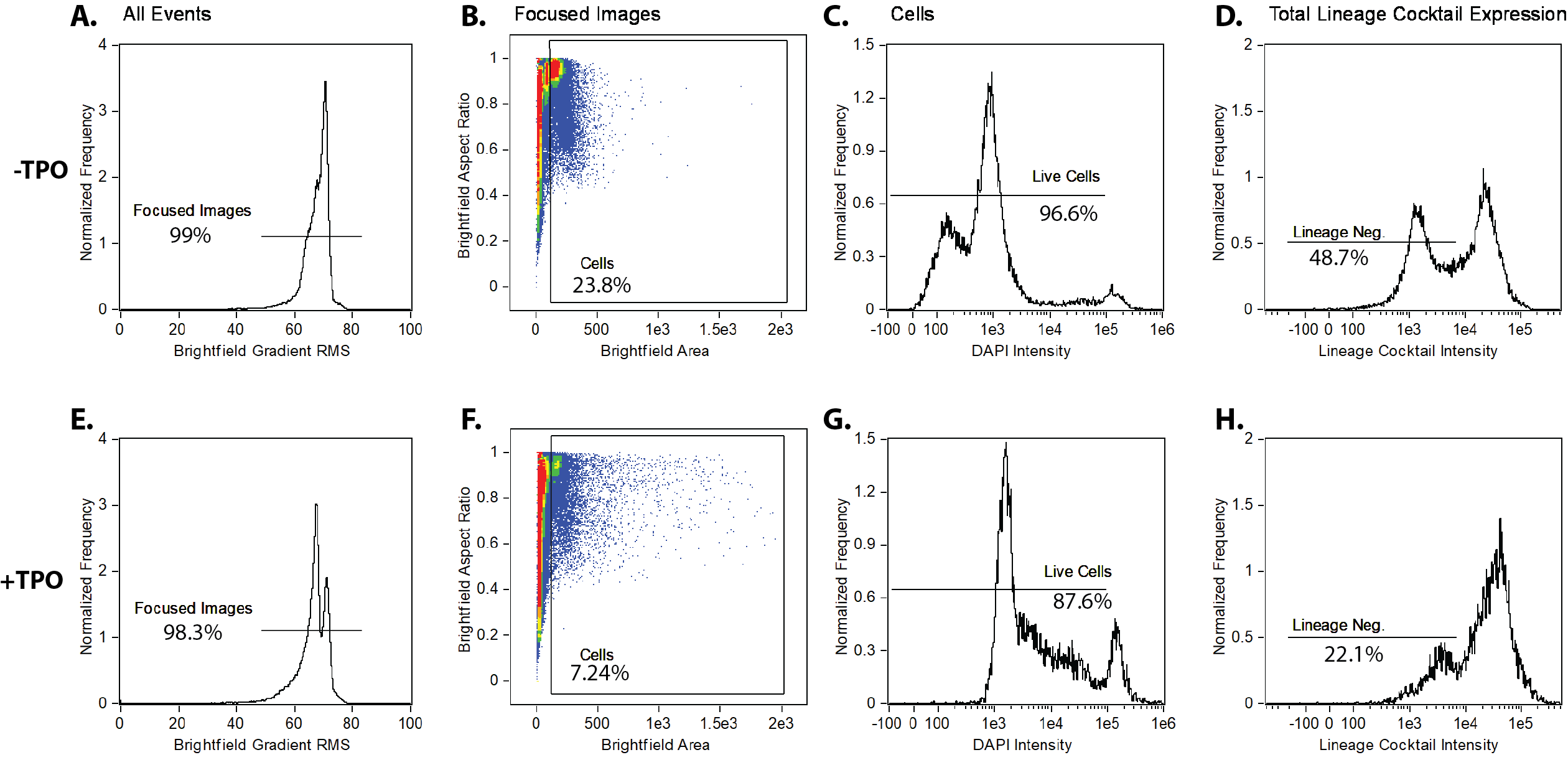


**Supplementary Figure 1. Gating strategy for Figure characterizing progenitor cell subpopulations. A.** Histogram of all events recorded in the -TPO condition by the ImageStream^®^X Mark II Imaging flow cytometer, gated for focused images. **B.** Dot plot of focused images gated for -TPO cells. **C.** Histogram of -TPO cells gated on DAPI intensity to identify live cells. **D.** Histogram of live -TPO cells gated on lineage cocktail intensity with a lineage negative gate indicating the cells not stained with lineage cocktail markers. E. Histogram of all events recorded in the +TPO condition by the ImageStream^®^X Mark II Imaging flow cytometer, gated for focused images. F. Dot plot of focused images gated for +TPO cells. G. Histogram of +TPO cells gated on DAPI intensity to identify live cells. H. Histogram of live +TPO cells gated on lineage cocktail intensity with a lineage negative gate indicating the cells not stained with lineage cocktail markers.


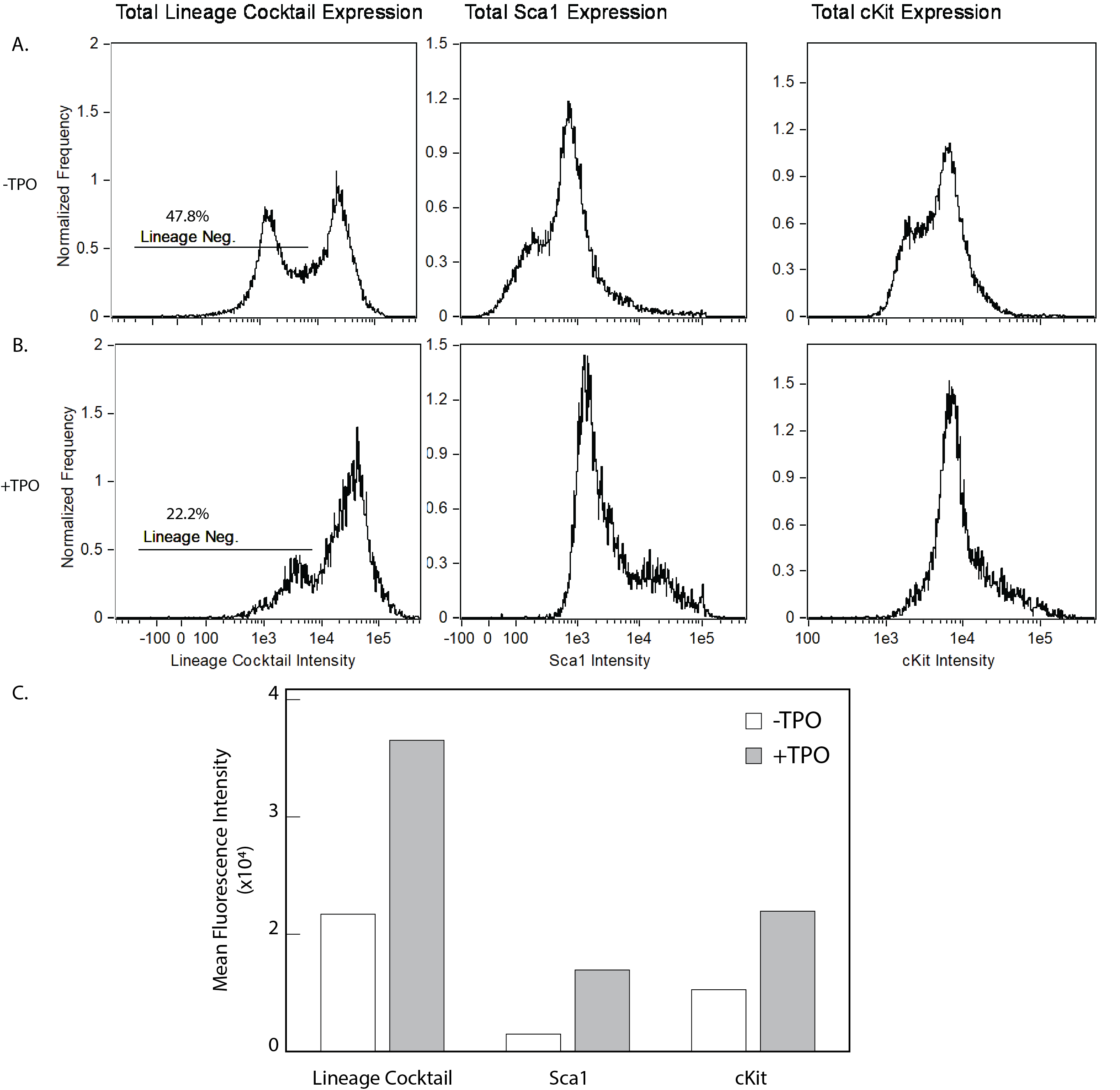


**Supplementary Figure 2. Total surface marker expression of bone marrow cells stained with lineage cocktail, Sca-1, and c-Kit before and after TPO exposure**. **A.** Mean fluorescence intensities (MFI) of lineage cocktail, Sca-1, and c-Kit antibody staining of -TPO bone marrow cells directly isolated from the mouse. **B.** MFI of lineage cocktail, Sca-1 and c-Kit antibody staining of +TPO bone marrow cells **C.** Bar graph summarizing the data in **A-B**.


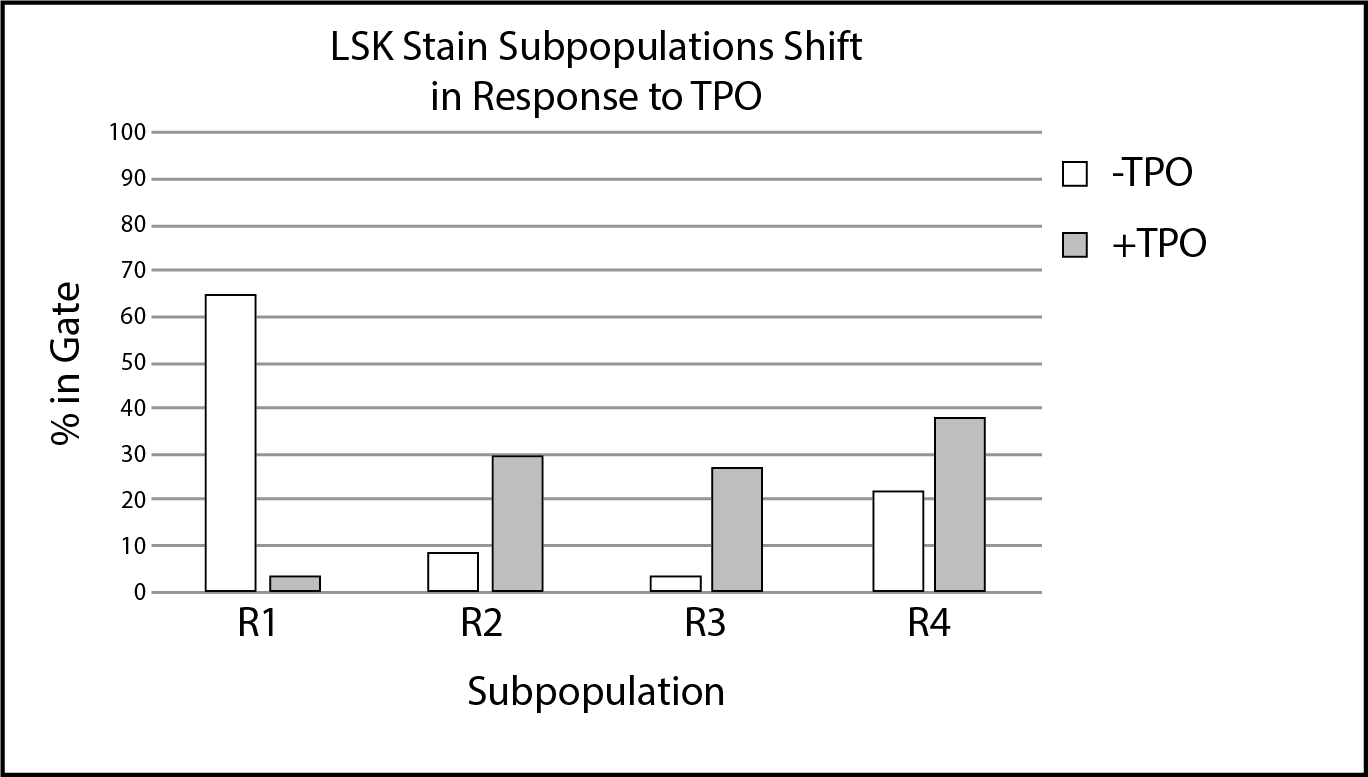


**Supplementary Figure 3. Quantification of lineage negative cells in each subpopulation**. Percent of gated cells in R1-R4 subpopulations from data in Figure 1. The percentage of cells in R1 decreases, while the percentage of cells in R2-R4 increases, respectively, in response to culturing with TPO.


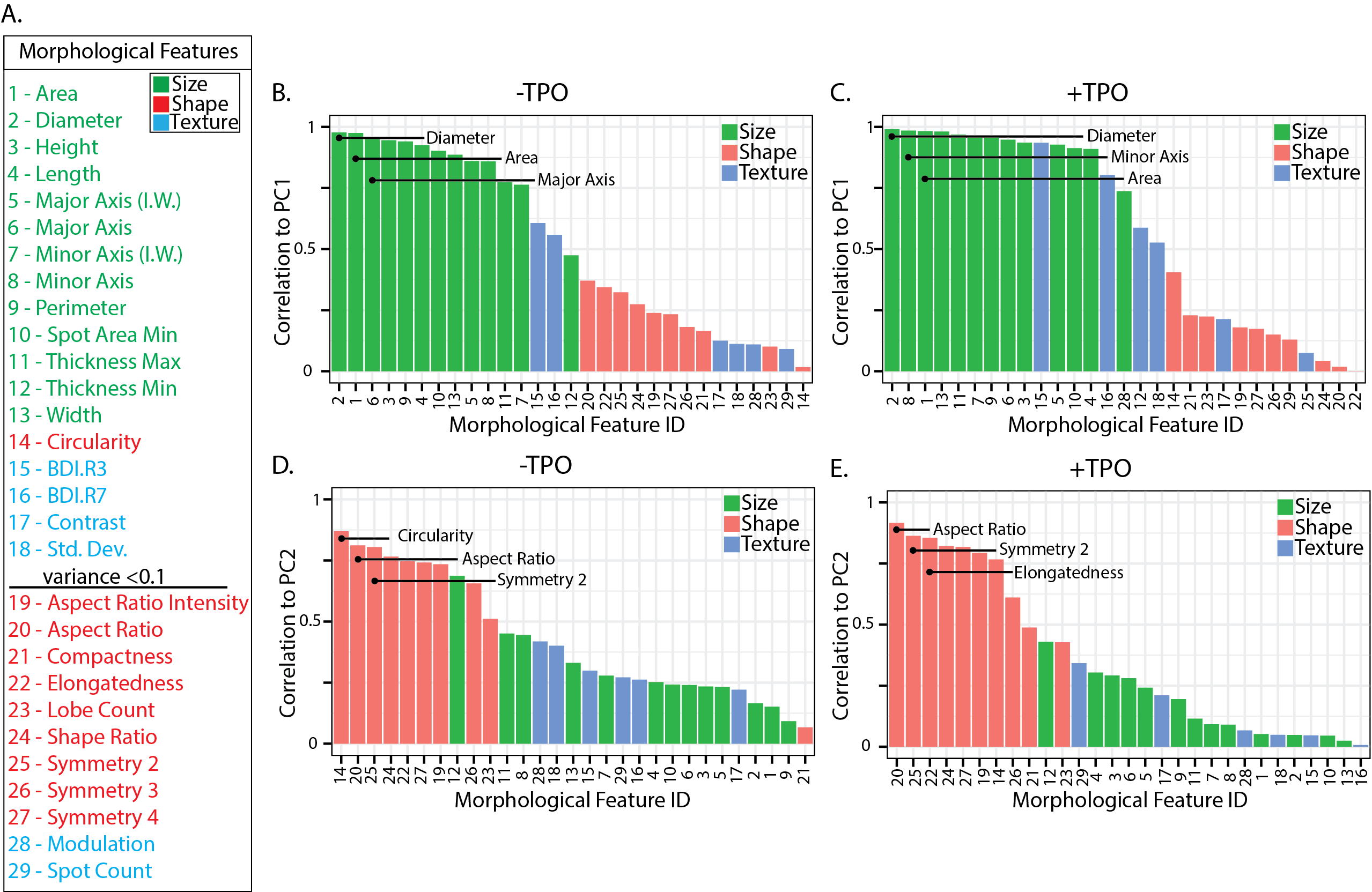


**Supplementary Figure 4. Morphological PCA Data. A**. Morphological feature list ID. Color indicates feature type: size (green), shape (red), and texture (blue). **B.** The absolute contribution/correlation of each morphological feature to PC1 D. and PC2 -TPO **C.** The absolute contribution/correlation of each morphological feature to PC1 +TPO and **E.** PC2 +TPO using the full set of morphological features. The top 3 most highly correlated features are listed for each principal component.


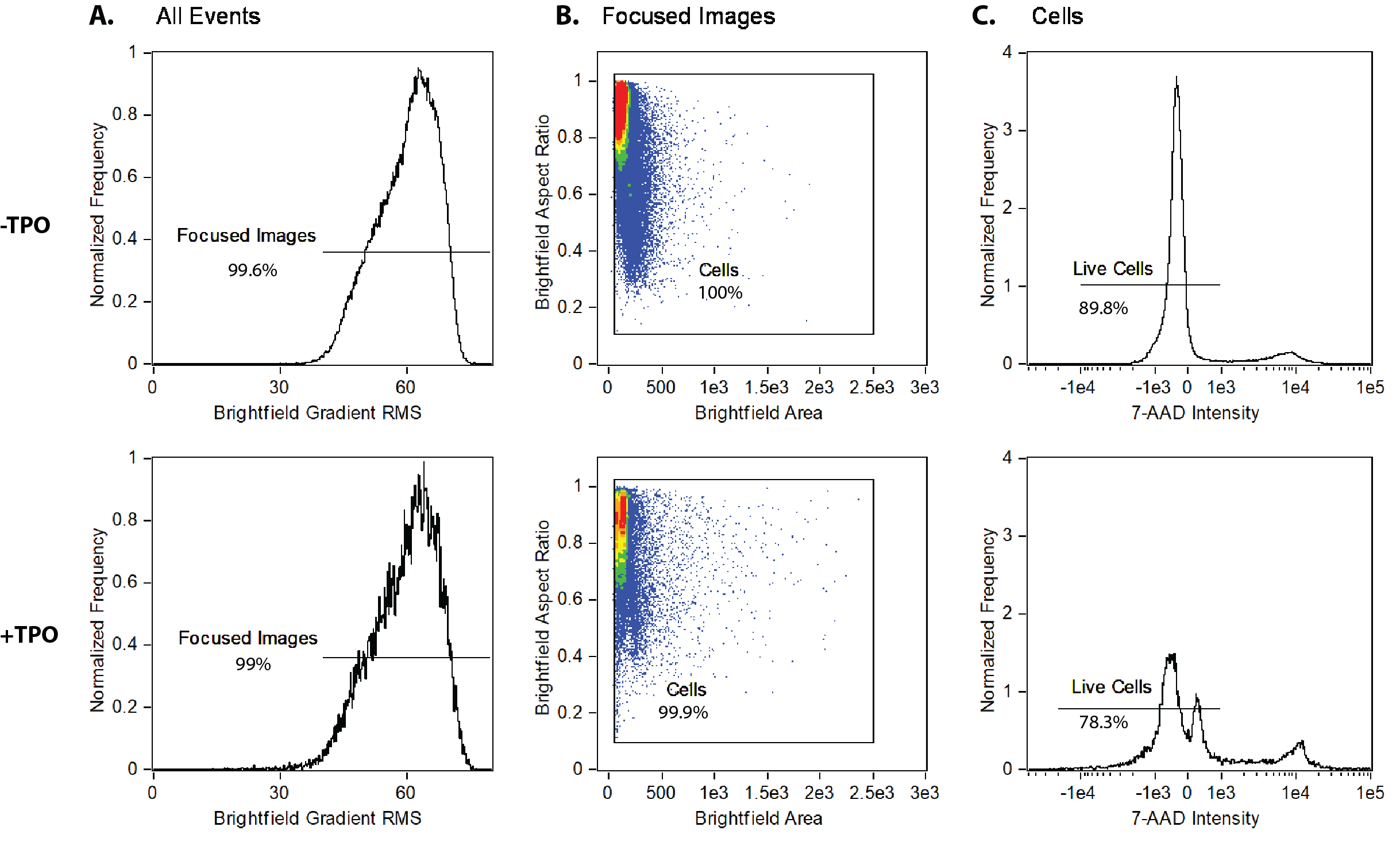


**Supplementary Figure 5. Gating strategy for classification of MK lineage subpopulations. A.** Histogram of all events recorded by the ImageStream^®^X Mark II Imaging flow cytometer, gated for focused images. **B.** Dot plot of focused images gated for cells. **C.** Histogram of cells gated on 7-AAD intensity to identify live cells. Top row: cells are stained immediately after isolation from the mouse bone marrow. Bottom row: cells cultured with 50 ng/mL TPO for three days.

**
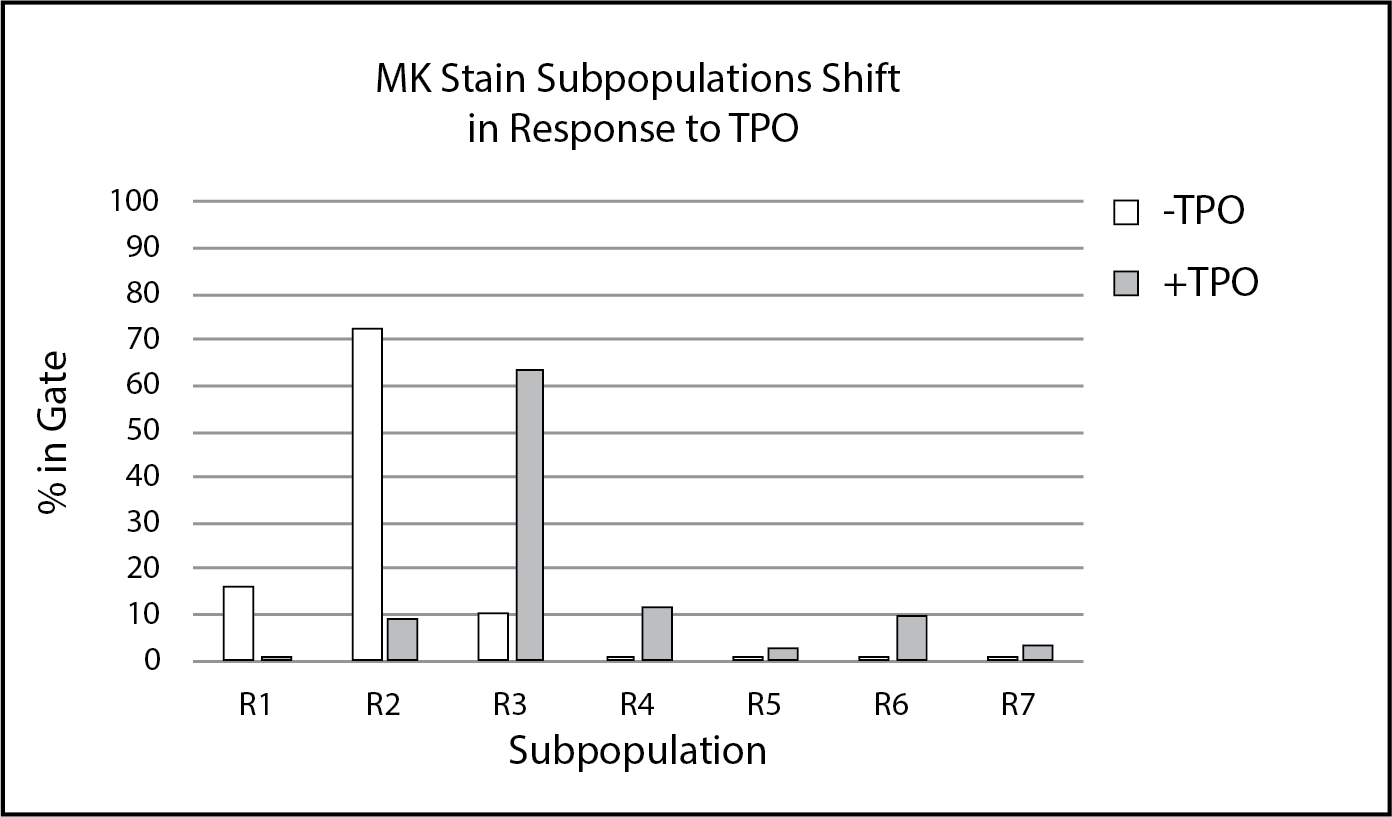
**

**Supplementary Figure 6. Quantification of live cells in each subpopulation.** Percent of gated cells in R1-R7 subpopulations from the classification of MK lineage subpopulations. The percentage of cells in R1-R2 decreases, while the percentage of cells in R2-R7 increases, respectively, in response to culturing with TPO.


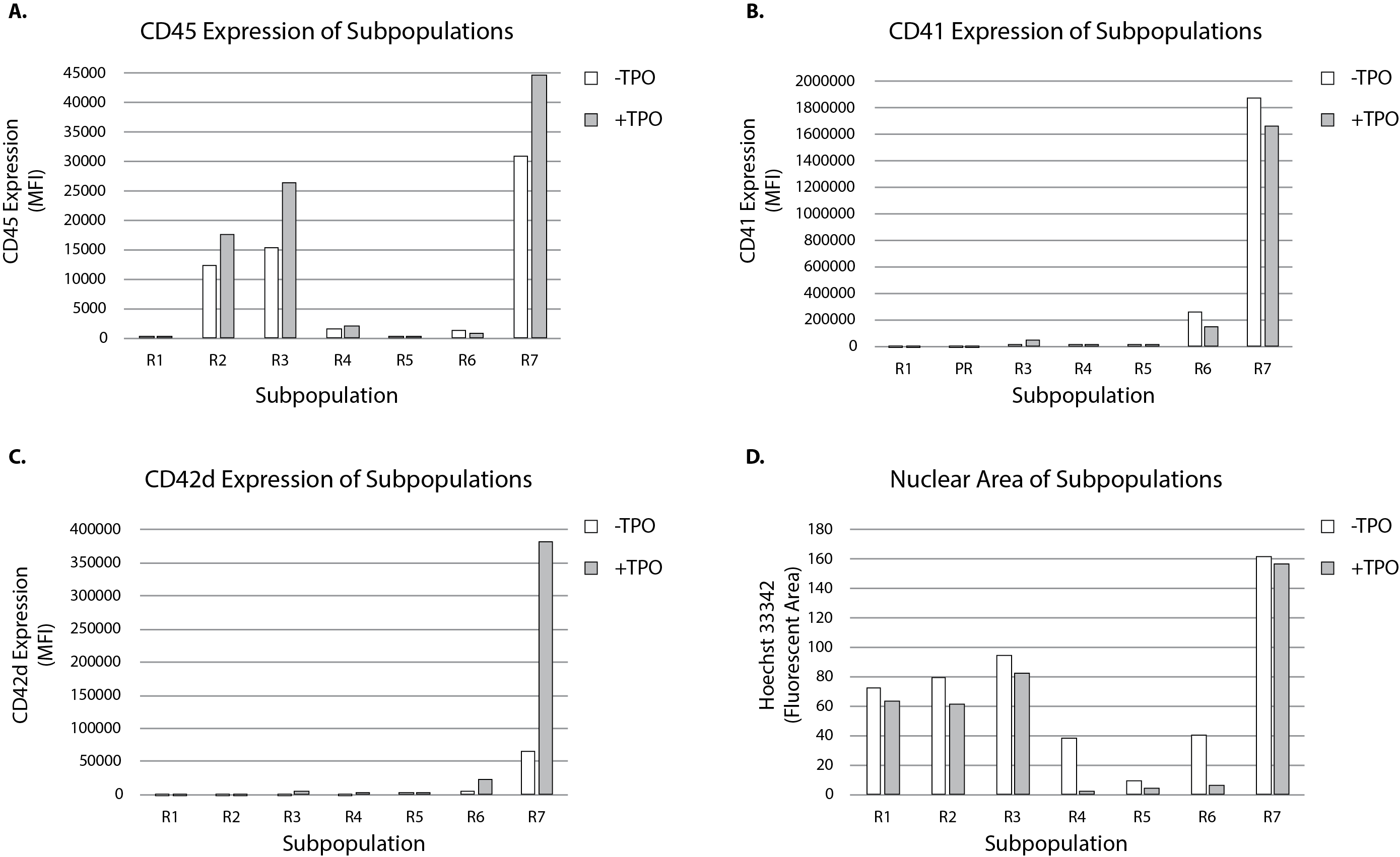


**Supplementary Figure 7. Analysis of surface marker expression and DNA content of subpopulations from the classification of MK lineage subpopulations. A.** MFI of CD45 positive cells in each subpopulation before and after TPO exposure. **B.** MFI of CD41 positive cells in each subpopulation before and after TPO exposure. **C.** MFI of CD42d positive cells in each subpopulation before and after TPO exposure. **D.** Nuclear area of cells in each population before and after TPO exposure.

**
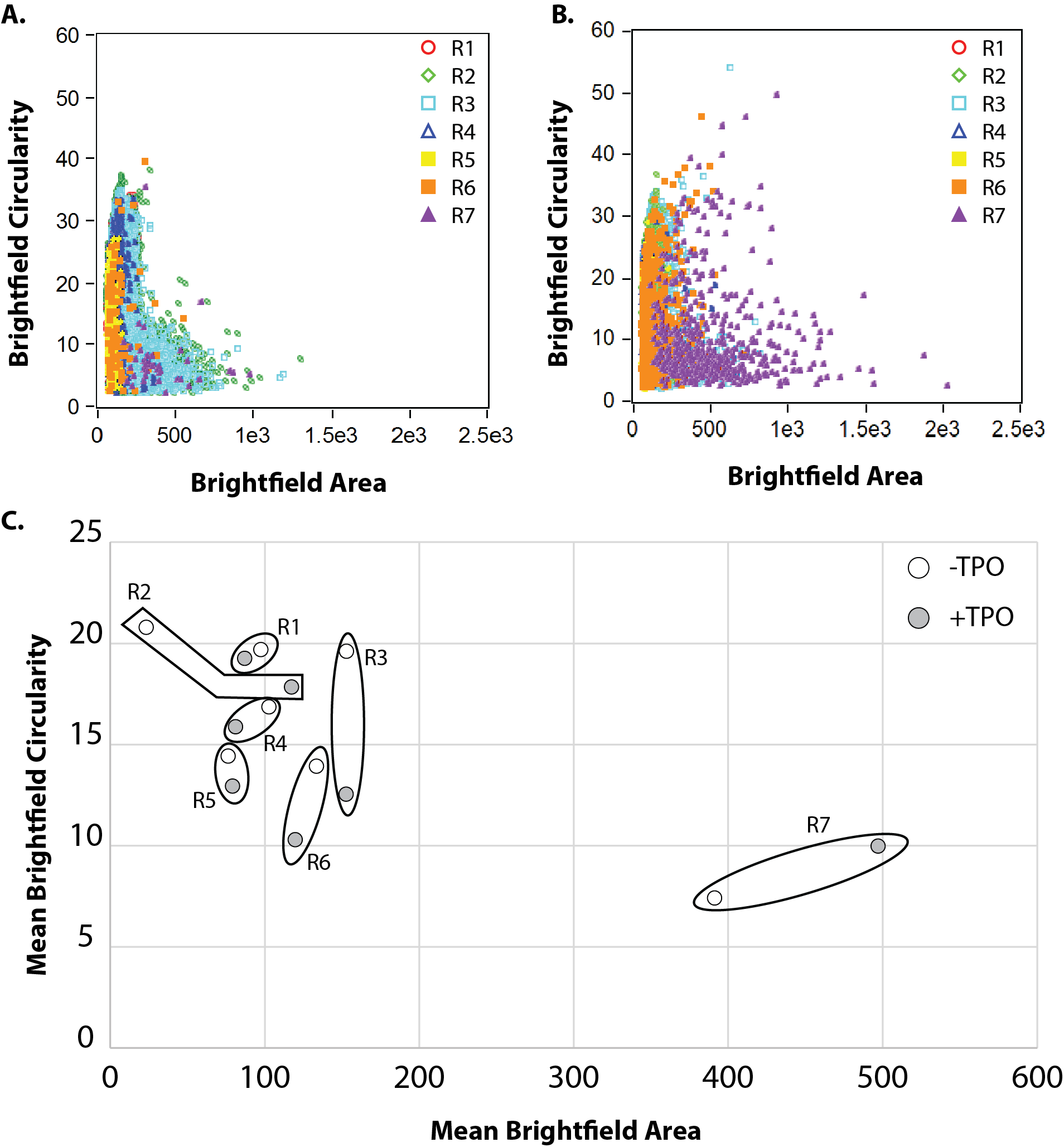
**

**Supplementary Figure 8. Brightfield circularity versus areas plots for each subpopulation enables identification of MKs by cell size alone. A.** Brightfield circularity vs. bright field area plot of cells directly isolated from the mouse bone marrow and not exposed to TPO. **B.** Brightfield circularity vs. bright field area plots of cells after culture with 50 ng/mL of TPO. **C.** Mean brightfield circularity vs. brightfield area of the different subpopulations for the two culture conditions.

**Supplementary Table**

**Table S1:** **Reagents used in flow cytometry experiments.** A comprehensive list of reagents used in each of the flow cytometry experiments, including reagent name, specificity/target information, brand, clone (if applicable), catalog and/or reference number, and dilution.

| **Key Resources Table** | | | | |
| --- | --- | --- | --- | --- |
| **Reagent Type (species) or resource** | **Designation** | **Source or Reference** | **Identifiers** | **Additional Information** |
| Antibody | PE-Cy™7 Rat anti-Mouse CD117 | BD Pharmingen | 561681 | Clone 2B8; Dilution used = 1:133 |
| Antibody | PE Rat Anti-Mouse Ly-6A/E | BD Pharmingen | 562059 | Clone D7; Dilution used = 1:100 |
| Antibody | APC Mouse Lineage Antibody Cocktail | BD Pharmingen | 51-9003632 | Dilution used = 1:5 |
| Antibody | CD45 Monoclonal Antibody, PE-Cyanine7, eBioscience™ | eBioscience | 25-0451-81 | Clone 30-F11; Dilution used = 1:133 |
| Antibody | PE Rat Anti-Mouse CD41 | BD Pharmingen | 561850 | Clone MWReg30; Dilution used = 1:100 |
| Antibody | CD42d Monoclonal Antibody, APC, eBioscience™ | eBioscience | 17-0421-80 | Clone 1C2; Dilution used = 1:50 |
| Antibody | BV421 Rat Anti-Mouse CD117 | BD Biosciences | 566290 | Clone  2B8; Dilution used = 1:80 |
| Antibody | BV510 Rat Anti-Mouse CD9 | BD Biosciences | 740128 | Clone KMC8; Dilution used = 1:80 |
| Antibody | CD90.1 (Thy-1.1) Monoclonal Antibody (HIS51), PerCP-Cyanine5.5, eBioscience™ | ThermoFisher Scientific | 45-0900-80 | Clone HIS51; Dilution used = 1:333 |
| Antibody | CD41a Monoclonal Antibody (eBioMWReg30 (MWReg30)), PE-Cyanine7, eBioscience™ | ThermoFisher Scientific | 25-0411-80 | Clone MWReg30; Dilution used = 1:60 |
| Antibody | CD127 Monoclonal Antibody (A7R34), APC-eFluor 780, eBioscience™ | ThermoFisher Scientific | 47-1271-80 | Clone A7R34; Dilution used = 1:80 |
| DNA Dye | DAPI | Thermo Scientific | 62247 |  |
| DNA Dye | Hoechst 33342 Solution | BD Pharmingen | 561908 | Dilution used = 1:1000 |
| DNA Dye | eBioscience™ 7-AAD Viability Staining Solution | eBioscience | 00-6993-50 |  |
| DNA dye | SYTOX™ Green Dead Cell Stain, for flow cytometry | ThermoFisher Scientific | S34860 |  |
| Equipment | ImageStream®X Mark II Imaging Flow Cytometer | Merck Millipore | NA |  |
| Equipment | BD FACSAria™ III | BD Biosciences | NA |  |
| Equipment | BD FACSCanto™ II | BD Biosciences | NA |  |
| Other | Fetal Bovine Serum | Gibco | 16000044 |  |
| Other | Penicillin Streptomycin | ThermoFisher Scientific | 15140122 |  |
| Other | IMDM Medium | ThermoFisher Scientific | 12440053 |  |
| Other | 26 G BD™ Needle 1/2 in. single use, sterile | BD | 305111 |  |
| Other | 35mm Corning™ TC-Treated Culture Dishes | Fisher Scientific | 08-772-20 |  |
| Other | ACK Lysis Buffer | ThermoFisher Scientific | A1049201 |  |
| Other | HBSS | Gibco | 14025092 |  |
| Other | Bovine Serum Albumin | ThermoFisher Scientific | BP1600-100 |  |
| Peptide, recombinant protein | Recombinant murine TPO | Peprotech | 315-14 |  |
| Software | IDEAS® | Amnis Corporation | NA |  |
| Software | FlowJo® | FlowJo, LLC | NA |  |
| Strain, (*Mus musculus*) | C57BL/6 | NA | NA |  |
